# Neuroprotective effects of walnut *(Juglans regia L.)* in nervous system disorders: A comprehensive review

**DOI:** 10.22038/ijbms.2024.79854.17302

**Published:** 2024

**Authors:** Mahboobeh Ghasemzadeh Rahbardar, Hossein Hosseinzadeh

**Affiliations:** 1 Pharmaceutical Research Center, Pharmaceutical Technology Institute, Mashhad University of Medical Sciences, Mashhad, Iran; 2 Department of Pharmacodynamics and Toxicology, School of Pharmacy, Mashhad University of Medical Sciences, Mashhad, Iran

**Keywords:** Analgesics, Anti-Inflammatory agents, Anti-oxidants, Epilepsy, Herbal medicine, Neurodegenerative diseases

## Abstract

Juglans regia L. (walnut) has a rich history in traditional medicine due to its various medicinal properties, including its neuroprotective effects on nervous system disorders. This updated review sheds light on the therapeutic potential of walnuts in nervous system disorders such as Alzheimer’s disease, Parkinson’s disease, depression, epilepsy, and pain, supported by evidence from in vivo and in vitro studies. These beneficial effects are attributed to the walnut’s rich composition of bioactive compounds, including gallic acid, protocatechuic acid, ferulic acid, sinapate, ellagic acid, p-hydroxybenzoic acid, p-coumaric acid, quercetin 3-galactoside, juglone, vanillic acid, quercetin, myricetin, kaempferol, apigenin, luteolin, daidzein, and others. The mechanisms underlying the neuroprotective effects of walnuts include decreasing oxidative stress, inflammation, apoptosis, proteolysis, β-amyloid plaque accumulation, acetylcholinesterase (AChE) activity, phosphorylated-c-Jun N-terminal kinase (p-JNK) levels, increasing adenosine triphosphate (ATP) levels, mitochondrial homeostasis, expression of mitophagy-related proteins, and activating the nuclear factor erythroid 2–related factor 2 (Nrf2)/Kelch-like ECH-associated protein 1 (KEAP1)/heme oxygenase-1 (HO-1) pathway. Although walnuts hold great promise in managing nervous system disorders and their complications, further preclinical and clinical investigations are necessary to consolidate these findings. This comprehensive review highlights the potential of walnuts as a natural therapeutic agent and encourages future research to unlock their full neuroprotective potential.

## Introduction

In neurodegenerative diseases, the central nervous system is damaged, resulting in sensory impairment or functional loss (1). These disorders, linked to various multifactorial etiologies, have resulted in social, financial, and medical burdens in recent years (2). Neuronal degeneration is caused by pathologic processes such as inflammation, oxidative stress, apoptosis, mitochondrial malfunction, and genetics (3-5). Disability, aging, and mortality are pathological markers of neurodegenerative disorders, including Alzheimer’s disease, Parkinson’s disease, and multiple sclerosis (6). Natural compounds and herbal medications have been utilized for medicinal purposes for a long time, dating back centuries (7-10). Moreover, researchers have focused increasingly on herbs in developing medicines in recent decades since they might have fewer side effects (11-13). Hence, in response to increased demand, pharmacological and therapeutic research has been expanding worldwide (14, 15). 

**Figure 1:**
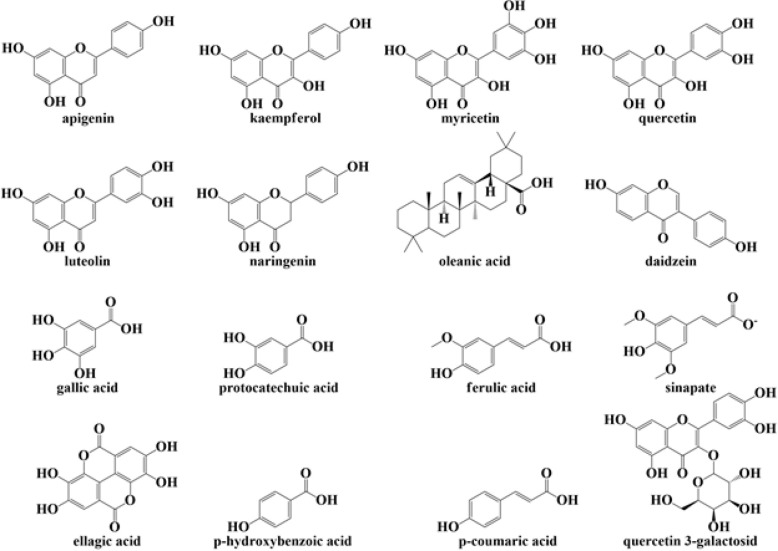
Chemical structures of some of the main constituents of walnut

*Juglans regia* L. (walnut), native to Southeastern Europe, Eastern Asia, and Northern America, is the most widely distributed tree nut in the world (16-18). Since ancient times, walnuts have been employed in human nutrition worldwide. The fruit’s seed section (the kernel) is consumed fresh, toasted, or blended with other confectionaries. Walnuts are used alone or in combination with almonds, dates, and raisins in a Middle Eastern pastry dish (19). Walnut is a nutrient-dense “brain food” since it is high in unsaturated fatty acids (20), proteins (21), polyphenols (22), and minerals (23). Flavonoids (kaempferol, myricetin, quercetin), flavons (apigenin, luteolin), flavonone (hesperetin, naringenin), isoflavonoid (daidzein), sterols, steroid (stigmasterol, campesterol, stigmast-5-en-3β,7α- diol, sitosterol), flavanol (gallocatechin), terpenoid (oleanolic acid, 3-alpha-corosolic acid, ursolic acid, 3-epikatonic acid), pectic compounds, phenolic acids (gallic acid, protocatechuic acid, ferulic acid, sinapate, protocatechuic acid derivative, ellagic acid, p-hydroxybenzoic acid, p-coumaric acid, quercetin 3-galactoside), and related polyphenols are also abundant in them (19, 24) (Figure 1). Unsaturated fatty acids derived from walnuts have been shown to possess nutritional benefits such as reducing blood cholesterol and providing anti-oxidant protection (25). The ability of walnut protein hydrolysates to scavenge free radicals and prevent lipid peroxidation has been documented (26, 27). Walnut proteins have also been shown to be a good source of bioactive peptides that have anti-oxidant, hypocholesterolemic, and angiotensin-converting enzyme inhibitory properties (27, 28). Gel chromatography was used to extract cancer cell growth inhibitory peptides from walnut protein hydrolysates, and these peptides were found to trigger apoptosis in cancer cells (29, 30).

Walnut leaves have been used for their antibacterial, anthelmintic, astringent, keratolytic, antidiarrheal, hypoglycemic, depurative, tonic, and carminative properties and the treatment of sinusitis, colds, and stomach aches in traditional medicine around the world (19, 31). Fresh leaves are applied to the naked body or the forehead to reduce fever or to swollen joints to relieve rheumatic pain in Turkish folk medicine (32). In Iranian traditional medicine, the kernel of *J. regia* has been used to treat inflammatory bowel disease (33). It is used in Palestine to treat diabetes, asthma, and prostate and vascular disorders (34). Walnut is a popular nut because it has several essential functional and physical properties, including anti-inflammatory and anti-oxidant activities (35-37), antidiabetic (38), antinociceptive (39), cancer prevention (40, 41), cholesterol reduction (42, 43), cardiovascular risk reduction (44), and cognitive enhancement (45, 46).

This study aims to comprehensively explore the pharmacological effects of walnut in treating and managing nervous system disorders, thereby attracting scientists’ attention to its therapeutic potential. While some previous studies have investigated the neuroprotective properties of walnuts, this review focuses specifically on *J. regia*, aiming to provide unique insights into its efficacy and distinct contributions. Also, the present comprehensive analysis delves into the specific benefits of *J. regia*, offering novel perspectives on its therapeutic applications. By categorizing the evidence based on *in vitro*, *in vivo*, and clinical trials, we aim to provide a comprehensive understanding of the effects of *J. regia* in various contexts of nervous system disorders.

Moreover, this study encompasses a wide range of neurological conditions, including learning and cognitive disorders, Parkinson’s disease, depression, anxiety, mood states, pain, and epilepsy. Our research specifically explores the neuroprotective effects of *J. regia*, highlighting its distinct properties and potential therapeutic applications. Through this examination, we seek to bridge the gap between basic science discoveries and their translation into effective clinical interventions.

To facilitate the accessibility and usability of our findings, we present two detailed tables within this article, offering a comprehensive overview of the studies discussed. These tables serve as valuable resources for researchers and clinicians, consolidating the evidence and facilitating a deeper understanding of the neuroprotective effects of *J. regia* in the context of nervous system disorders.

By highlighting this study’s unique contributions and novel insights, we aim to inspire further research and encourage the exploration of *J. regia* as a potential therapeutic agent in neuroprotection. Ultimately, we strive to connect the value of *J. regia* from the basic sciences to its potential practical implications in patients’ beds.

## Methods

**Figure 2:**
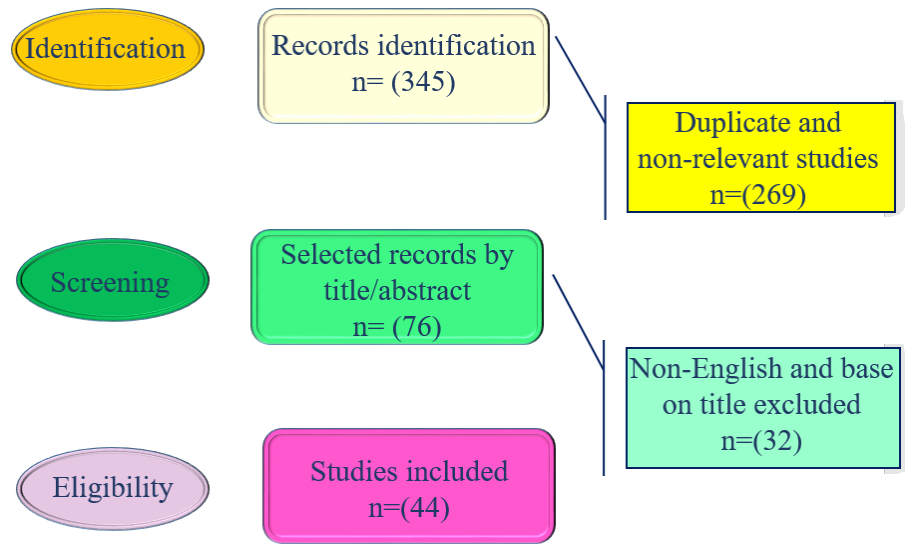
Chart of the search strategy

Scopus, Google Scholar, and PubMed were among the databases used in this research. All publications (*in vitro*, *in vivo*, and clinical trials) were collected until 2023 (Figure 2). The following terms were used in the search: “*Juglans regia* L.”, “walnut”, “nervous system”, “depression”, “memory”, “Alzheimer’s disease”, “epilepsy”, “seizure”, “anticonvulsant”, “Parkinson’s disease”, “anxiety”, “mood”, “pain”, “neuropathic pain”, “antinociceptive”, “analgesic”, and “Alzheimer’s disease”.

In relation to the criteria for inclusion and exclusion, our team took into account articles that satisfied the following conditions:

• Articles that examined the neuroprotective effects of walnuts on learning and cognitive disorders, Parkinson’s disease, depression, anxiety, mood states, pain, and epilepsy.

• Studies using *in vitro* models, animal models, or human subjects. 

• Articles written in the English language.

• Articles published in peer-reviewed academic journals. 

Our team disqualified the following types of items:

• Articles were written in languages other than English.

• Studies that did not specifically address the mentioned disorders or the effects of walnuts.

• Articles that were not peer-reviewed, including conference abstracts, editorials, and letters.

**Neuroprotective effects of** ***J. regia*** **against nervous system disorders**

***Learning and cognitive disorders***

The brain’s learning and memory functions are surprisingly complicated cognitive processes. Cognitive functions rapidly form throughout development, become stable in adults, and may diminish with age (47). Cognitive dysfunction, dementia, and Alzheimer’s disease are on the rise worldwide, with developing countries markedly affected. Dementia affected 35.6 million people globally in 2010 and is expected to rise to 65.7 million in 2030 and 115.4 million in 2050 (48). 

Traditional neurotransmitter systems, such as cholinergic and glutamatergic, are important for memory and learning. For example, various molecular and pharmacological evidence shows that cognitive impairment is caused by the stimulation of the glutamatergic system and a deficit in cholinergic neurotransmission (49). Moreover, the excessive deposition of β-amyloid plaques in the hippocampus has been linked to Alzheimer’s disease-related cognitive impairment. In addition, the gut-brain axis has been identified to play a role in various neurological disorders, including Alzheimer’s disease (50). Furthermore, mitochondrial dysfunction and chronic inflammation appear to play essential roles in cognitive decline and impaired motor function in physiological aging, as well as neurodegenerative diseases such as Alzheimer’s disease. Changes in the oxidative phosphorylation system, which result in lower complex activity, depolarization of the mitochondrial inner membrane space and matrix, and hence reduced adenosine triphosphate (ATP) generation, are all hallmarks of mitochondrial dysfunction (51). In conjunction with amplified oxidative stress and inflammation, a damaged neuronal housekeeping function is recognized as autophagy, a process to mitigate the increase of toxic or misfolded polyubiquitinated proteins in the brain. Dysfunctional autophagy has also been associated with neurodegenerative diseases, including Alzheimer’s (52). Hence, developing innovative medicines for regaining cognitive function with minimal adverse effects has sparked a lot of attention.

***In vitro***

An investigation illustrated that treating PC12 cells with walnut extract could reduce β-amyloid-mediated cell death, lactate dehydrogenase release, apoptosis, deoxyribonucleic acid (DNA) damage, and reactive oxygen species (ROS) generation (53). A recent study disclosed that treating SH-SY5Y cells exposed to rotenone with a lipophilic walnut extract boosted ATP levels, citrate synthase activity, and neurite growth and decreased peroxidase activity and amyloid-β _1–40 _(54).

***In vitro*** **plus** ***in vivo***

**Figure 3:**
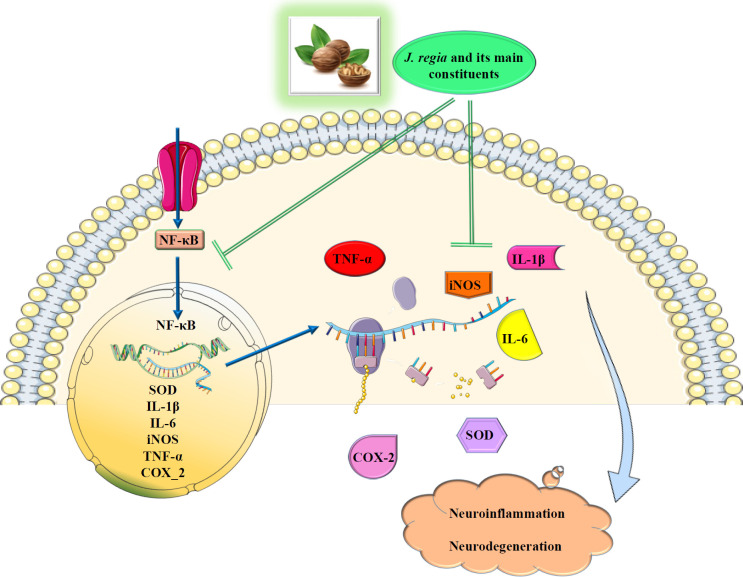
The proposed mechanism of the neuroprotective action of walnuts and their main components (Images from https://smart.servier.com)

The obtained data from an investigation showed that treating PC12 cells with hydrolysates from walnuts reduced nonviable apoptotic cells, and the *in vivo* part of the study demonstrated that oral administration of hydrolysates from walnuts improved memory and consolidated memory ability in mice (55). Another study claimed that treating PC12 cells with walnut protein hydrolysates caused an increase in superoxide dismutase (SOD) and glutathione peroxidase (GPx). The apoptosis rate, ROS production, Ca^2+^ influx, and mitochondrial membrane potential collapse were also decreased in PC12 cells. The *in vivo* part of this study revealed that administration of walnut protein hydrolysates to rats with sleep deprivation-induced memory impairment enhanced behavioral performance, catalase (CAT), GPx, and SOD levels, and attenuated the malondialdehyde (MDA) level of the hippocampus (56). It has been reported that treating PC12 cells with a peptide from walnut reduced H_2_O_2_-induced cell death. This compound could also amplify the messenger ribonucleic acid (mRNA) expression level of brain-derived neurotrophic factor and crossing times in a zebrafish model and attenuate caspase 3, 7, and 8 activities, glial cell line-derived neurotrophic factor and Bcl-2 Associated X (Bax) mRNA expression levels, escape latency, and memory impairments (20). The results of an earlier study revealed that exposing HEK-293-E22G cells and mice to a bioactive peptide (PW5) derived from walnut improved cognition by lowering the formation of β-amyloid plaques in the brain. Furthermore, the metabolomic analysis showed that serum norepinephrine and isovalerate levels were significantly higher in response to the PW5 intervention, while serum levels of acetylcholine (ACh) and valerate were significantly lower. According to 16s ribosomal ribonucleic acid (rRNA) studies, PW5 improves gut dysbiosis in APP/PS1 transgenic mice by boosting the relative abundance of Firmicutes while lowering Proteobacteria and Verrucomicrobia (50). It has also been reported that treating BV-2 cells exposed to lipopolysaccharide with walnut peptides could remarkably increase mitochondrial homeostasis and lower pro-inflammatory mediators, cytokines, and ROS levels. The results of the *in vivo* section of the research revealed that administration of walnut peptides to mice with cognitive impairment increased SOD, GPx, and CAT levels. It also attenuated MDA levels and inflammation markers such as prostaglandin E2 (PGE2), interleukin (IL)-6, IL-1β, and tumor necrosis factor-alpha (TNF-α) in the brain (57) (Figure 3). Using an *in vitro* acetylcholinesterase (AChE) inhibition assay, acrylamide-induced neurotoxicity in zebrafish larvae, and scopolamine-induced cognitive deficit in adult zebrafish, the efficacy of an unidentified anthraquinone (1-Hydroxy-5,5-dimethyl-5,6,7,8-tetrahydro-9,10-anthraquinone) from *J. regia* was investigated against cognitive deficits. The results revealed that this compound demonstrated strong AChE inhibition, reversed acrylamide-induced neurotoxicity, and enhanced learning and memory abilities (47). Another study indicated that treating HT-22 cells and mice with learning and memory impairment with walnut-derived peptide (YVLLPSPK) enhanced the expression of mitophagy-related proteins, activated the nuclear factor erythroid 2-related factor 2/Kelch-like ECH-associated protein 1/heme oxygenase-1 (NRF2/KEAP1/HO-1) pathway, PTEN-induced putative protein kinase 1 (PINK1)-mediated mitophagy, and decreased oxidative stress (58).

The extensive investigation into the effects of walnut-derived compounds on cellular and cognitive function reveals promising therapeutic potential. Studies have demonstrated that hydrolysates and peptides from walnuts exhibit neuroprotective properties by reducing apoptosis and oxidative stress and improving memory abilities in both *in vitro *and* in vivo* models. These compounds have been shown to enhance anti-oxidant enzyme activity, mitigate mitochondrial dysfunction, and modulate neurotrophic factors, leading to improved cognitive function and behavioral performance. Additionally, walnut-derived peptides have shown efficacy in ameliorating neuroinflammation, reducing β-amyloid plaque formation, and improving gut dysbiosis associated with cognitive impairment. Moreover, identifying an anthraquinone from walnuts as a potent acetylcholinesterase inhibitor with neuroprotective effects further underscores the potential of walnut-derived compounds in treating cognitive deficits. These findings highlight the importance of exploring natural sources like walnuts to develop novel therapies to combat cognitive decline and neurodegenerative diseases. Further research is warranted to elucidate the underlying mechanisms and optimize the therapeutic potential of these compounds for clinical applications.

***In vivo***

It has been shown that feeding aged rats with walnuts significantly increased autophagy, autophagy-related 7 (ATG7), and Beclin 1, decreased polyubiquitinated protein aggregation, phosphorylation of the mammalian target of rapamycin (mTOR), inflammation**,** and oxidative stress in the striatum and hippocampus (52). An *in vivo* study evaluated the effect of walnut suspension on scopolamine-induced memory impairment in rats. The data indicated that the prescription of walnut could significantly improve memory by increasing ACh concentration in the frontal cortex and hippocampus, increasing SOD, CAT, and GPx levels, and lowering AChE activity as well as MDA levels (59). It has also been found that adding walnuts to the diet of mice with Alzheimer’s disease improved the function of anti-oxidant enzymes and decreased oxidative stress, ROS production, protein oxidation, and lipid peroxidation. The authors stated that the administration of walnuts in the long term was more beneficial in decreasing oxidative stress (60). Another study claimed that adding walnut kernel powder to the diet of mice with memory deficits could markedly amend the disorder by augmenting ACh levels and attenuating cholinesterase activity in the brain and total cholesterol levels (61). It has been shown that adding walnut kernel to the diet of rats with age-related cognitive dysfunction augmented hippocampal neurogenesis, hippocampal phosphorylated cyclic adenosine monophosphate response element-binding protein (p-CREB) and brain-derived neurotrophic factor (BDNF) expression, fundamental intracellular molecules associated with hippocampal neurogenesis, besides reducing spatial memory loss, locomotor activity deficiency, and recognition behavior reduction (62). The feeding of the walnut kernel and septum to aged rats caused a significant increase in anti-oxidant activity and decreased ROS, nitric oxide levels, AChE activity, and the onset of aging processes (63). It has also been shown that receiving walnut extract in mice regulated blood-brain barrier (BBB) function and augmented mitochondrial function, SOD, ACh, and ATP levels. The walnut extract also decreased MDA levels, AChE activity, TNF-α, phosphorylated c-Jun N-terminal kinase (p-JNK), IL-1β levels, behavioral dysfunction, and memory deficit (64). Another *in vivo* study assessed the effect of walnut oil on mice with memory dysfunction. It was observed that walnut oil could pointedly enhance choline acetyltransferase and SOD activities, glutathione (GSH) levels, and reduce memory impairment, AChE activity, and MDA levels in the brain, as well as histological alterations of neurons in the CA1 and CA3 regions of the hippocampus (65). It has been disclosed that adding walnuts to mice’s diet improved spatial memory, increased hydroxy-polyunsaturated fatty acids in the brain, and declining arachidonic acid-based oxylipin levels (51). Furthermore, a recent *in vivo* study illustrated that receiving walnut-derived peptide (WNP-10) in mice with memory deficits improved learning and memory capability by increasing phosphorylation of phosphatidylinositol-3-phosphate 5-kinase, preserving lysosome homeostasis, regulating N-acetylgalactosamine 6-sulfate sulfatase, cathepsin L, phosphatidylinositol-3-phosphate 5-kinase, and AP-3 complex subunit mu-1 expression. It was also observed that the proteome of WNP-10-treated mice had 88 differentially expressed proteins compared to the scopolamine-induced impairment group (66).

The comprehensive data from various studies underscores the potential of walnuts and their derivatives in ameliorating cognitive deficits and age-related cognitive decline. Feeding aged rats with walnuts led to significant increases in autophagy markers, decreased protein aggregation, and reduced inflammation and oxidative stress in brain regions associated with memory function. *In vivo* studies further demonstrated that walnut supplementation improved memory impairment induced by scopolamine, Alzheimer’s disease, and aging by increasing acetylcholine levels, anti-oxidant enzyme activity, and regulating neurogenesis-related molecules. Moreover, walnut extract and oil were found to regulate BBB function, enhance mitochondrial function, and decrease inflammatory markers and lipid peroxidation. Additionally, walnut-derived peptides showed promising effects in improving learning and memory capabilities by modulating lysosome homeostasis and the expression of various proteins involved in memory formation. These findings highlight the potential of walnuts as a dietary intervention for promoting cognitive health and warrant further exploration into their mechanisms of action and clinical applications.

**Clinical trials**

A clinical trial evaluated the effect of two years of consumption of walnuts in older adults, and their findings showed a delayed cognitive decline but no effect on cognition (67). The contradiction between the results obtained from this study and those of other clinical trials might be explained by the fact that some participants in this study were smokers, and the authors stated that some individuals had lower baseline neuropsychological test scores than others; thus, the inconsistency of the samples might affect the results. Moreover, it has been reported that whole walnut intake for four years in older American adults revealed neuroprotective effects, was positively linked with health behaviors and socioeconomic status, and increased cognitive scores (68). Furthermore, in a recent clinical trial on teenagers and older people, the administration of walnuts led to increased adult intelligence scale and sleep quality index in both older adults and teenagers. Treatment with walnuts also boosted average scores for test subjects in English, mathematics, and Chinese examinations in teenagers (69) (Table 1).

These studies indicate that walnut kernel, septum, different kinds of walnut extracts, as well as hydrolysates, peptide, polyphenol, and oligopeptide from walnut can ameliorate learning and memory performance via decreasing oxidative stress (by attenuating MDA and ROS levels, and increasing ROS, SOD, CAT, GSH, and GPx), inflammation (through lowering PGE2, IL-6, IL-1β, and TNF-α), apoptosis (via reducing lactate dehydrogenase release, Ca^2+^ influx, mitochondrial membrane potential collapse, DNA damage, activity of caspases 3,7, and 8, mRNA expression levels of Bax), proteolysis (by decreasing polyubiquitinated proteins aggregation), β-amyloid plaques accumulation, mRNA expression levels of glial cell line-derived neurotrophic factor, phosphorylation of mTOR in striatum and hippocampus, AChE activity, p-JNK amounts, histological alterations of neurons in CA1 and CA3 regions of hippocampus, besides increasing ATP level, citrate synthase activity, neurite growth, mRNA expression level of BDNF, serum norepinephrine and isovalerate levels, mitochondria homeostasis, expression of mitophagy-related proteins and activated the NRF2/KEAP1/HO-1 pathway, PINK1-mediated mitophagy, autophagy, ATG7 and Beclin 1, hippocampal neurogenesis, hippocampal p-CREB and BDNF, expressions of anti-oxidant defense-related protein, BDNF, CREB, AChE and Keap1 inhibitors, and phosphorylation of phosphatidylinositol-3-phosphate 5-kinase. Moreover, long-term walnut supplementation was shown to be more effective in lowering oxidative stress in Alzheimer’s disease. It also offers walnut benefits as a whole and advances its molecular effects, from anti-oxidant and anti-inflammatory benefits to neuronal housekeeping. Therefore, it might be concluded that a walnut-rich diet may assist in lessening the risk or postponing the beginning and progression of Alzheimer’s disease. Moreover, walnuts have the potential to become a new nutritional intervention for youth and older adults, improving memory and cognitive performance.

The cumulative evidence from various clinical trials suggests that walnut consumption may positively impact cognitive function across different age groups. While some studies have reported no significant effects on cognition in older adults, others have demonstrated improved cognitive scores and an increased intelligence scale and sleep quality index in both teenagers and elderly individuals. The neuroprotective effects of walnuts are likely mediated by their bioactive compounds, including hydrolysates, peptides, polyphenols, and oligopeptides. These compounds have been shown to reduce oxidative stress, inflammation, apoptosis, proteolysis, and β-amyloid plaque accumulation while enhancing mitochondrial function, autophagy, and neurogenesis. Long-term walnut supplementation appears particularly beneficial in lowering oxidative stress in Alzheimer’s disease, suggesting its potential as a nutritional intervention to delay the onset and progression of cognitive decline. Overall, the findings highlight the potential of walnuts as a dietary strategy to improve memory and cognitive performance across different age groups.

**Figure 4:**
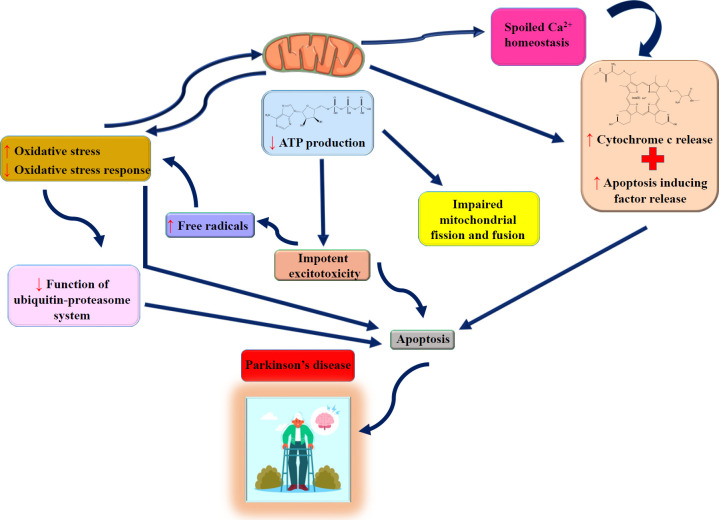
The roles of mitochondrial damage, oxidative stress, and apoptosis in Parkinson’s disease pathology (Images from https://smart.servier.com and https://www.freepik.com)

**Table 1:** Effect of *Juglans regia* L. on learning and cognitive disorders

Compound	Study design	Doses/Duration	Results	Ref.
*In vitro*				
Walnut extract	*in vitro, *PC12 cells	2 or 4 μg	↓ β-amyloid-mediated cell death, lactate dehydrogenase release, apoptosis, DNA damage, ROS generation	(53)
Lipophilic walnut extract	*in vitro, *SH-SY5Y cells	10 μg/ml	↑ ATP level, citrate synthase activity, neurite growth ↓ peroxidase activity, amyloid-β _1–40_	(54)
*In vitro* and *in vivo*				
Defatted walnut meal	*in vitro, *PC12 cells *in vivo, * mice	0.10, 0.25, 0.50 mg/ml, 24 hr 167, 333, and 1000 mg/kg, 40 days, PO	↑ antioxidant activity in PC12 cells ↓ apoptosis in PC12 cells ↑ learning and memory performance	(93)
Hydrolysates from walnut	*in vitro, *PC12 cells *in vivo, * mice	0, 0.05, 0.1, 0.2, 0.5, 1, 2, 5 mg/ml, 24 hr 333.3 mg/kg, 4 weeks, PO	↓ nonviable apoptotic cells in PC12 cells ↑ improved memory and consolidated memory ability	(55)
Walnut protein hydrolysates	*in vitro, *PC12 cells *in vivo, * rats	0.10 mM, 24 hr 666 mg/kg body, 20 days, PO	↑ SOD and GPx in PC12 cells ↓ apoptosis, ROS production, Ca^2+^ influx, and mitochondrial membrane potential collapse in PC12 cells ↑ behavioral performance, CAT, GPx, and SOD levels of the hippocampus ↓ MDA level	(56)
Peptide from walnut	*in vitro, *PC12 cells *in vivo, * zebrafish	0, 1, 10, 30, 100, and 200 μg/ml, 24 hr 30, 100, 300 mg/kg, 7 days, PO	↓ H_2_O_2_-induced cell death in PC12 cells ↑ mRNA expression level of BDNF significantly, more crossing times ↓ activity of caspases 3,7, and 8, mRNA expression levels of Bax and glial cell line-derived neurotrophic factor, escape latency, memory impairments	(20)
Pro-Pro-Lys-Asn-Trp (PW5)	*in vitro, *HEK-293-E22G *in vivo, * APP/PS1 transgenic mice	0.05 and 0.5 mM, 48 hr 80, 400 mg/kg, 12 weeks, PO	↑ serum norepinephrine and isovalerate levels, cognitive improvement ↓ β-amyloid plaque accumulation, serum levels of ACh and valerate	(50)
Walnut peptides	*in vitro, *BV-2 cells *in vivo, * mice	0.10 mM 666 mg/kg, 21 days, PO	↑ mitochondria homeostasis ↓ pro-inflammatory mediators and cytokines, ROS amount in BV-2 cells ↑ SOD, GPx, and CAT levels in the brain ↓ inflammation (PGE2, IL-6, IL-1β, and TNF-α) and oxidative stress in the brain	(57)
1-Hydroxy-5,5-dimethyl-5,6,7,8-tetrahydro-9,10-anthraquinone	*in vitro, *zebrafish larvae *in vivo, *adult zebrafish	15, 30 μM, for 72 hr 15, 30 μM	- inhibited AChE ↑ learning and memory abilities ↓ acrylamide-induced neurotoxicity	(47)
Walnut-derived peptide (YVLLPSPK)	*in vitro, *HT-22 cells *in vivo, *mice	100 μM), for 24 hr. 60 mg/kg, 4 weeks, PO	↑ expression of mitophagy-related proteins and activated the NRF2/KEAP1/HO-1 pathway ↑ PINK1-mediated mitophagy ↓ cognitive deficiency, oxidative stress	(58)
Gimcheon 1ho cultivar	*in vitro, *PC12 and HT22 cells *in vivo,* mice	20 and 50 μg/ml, 24 hr 20 and 50 mg/kg, four weeks, PO	↑ cell viability ↓ ROS production ↓ behavioral and memory dysfunction, lipid peroxidation, cholinergic system impairment, FRAP, AGEs	(94)
*In vivo*				
Walnut	*in vivo,* rats	6% or 9% of the diet, 15 weeks, PO	↑ autophagy, ATG7, and Beclin 1, ↓ polyubiquitinated protein aggregation, phosphorylation of mTOR, inflammation, and oxidative stress in the striatum and hippocampus	(52)
Walnut kernel	*in vivo,* pregnant rats	6% of diet during gestation and lactation, PO	↑ learning and memory of rat offsprings	(95)
Polyphenol extracts from walnut	*in vivo,* mice	200 μg/g body weight, eight weeks, PO	↑ number of crossings, brain SOD activity, learning and memory functions ↓ escape latency, swimming distance, brain MDA level	(96)
Walnut	*in vivo,* mice	6% or 9% of the diet, ten months, PO	↑ memory function, learning ability, motor development ↓ anxiety	(97)
Walnut	*in vivo,* rats	2%, 6%, and 9% of the diet, four weeks, PO	↓ memory impairments, AChE activity	(98)
Walnut protein hydrolysate	*in vivo,* mice	0.2, 0.33, 0.66 g/kg, five days, PO	↑ target times and crossing times in the spatial probe test, escape latency ↓ error times in the step-down avoidance test	(99)
Walnut suspension	*in vivo,* rats	200, 400, or 800 mg/kg, 4 weeks, PO	↑ ACh concentration in frontal cortex and hippocampus, SOD, CAT, and GPx amounts ↓ AChE activity, MDA level	(59)
Walnut	*in vivo,* mice	6% or 9% of diet, 5, 10, or 15 months, PO	↑ the function of antioxidant enzymes ↓ oxidative stress, ROS, protein oxidation, and lipid peroxidation	(60)
Walnut kernel powder	*in vivo,* mice	500 and 1000 mg/kg, 14 days, PO	↑ ACh level in the brain ↓ memory deficits, cholinesterase activity of the brain, total cholesterol amounts	(61)
Walnut kernel	*in vivo,* rats	6% and 9%, eight weeks, PO	↑ hippocampal neurogenesis, hippocampal p-CREB, and BDNF expression ↓spatial memory loss, locomotor activity deficiency, recognition behavior reduction	(62)
Walnut kernel and septum	*in vivo,* rats	9% of the daily diet given, 56 days, PO	↑ antioxidant activity ↓ ROS and nitric oxide levels, AChE activity, onset of aging processes	(63)
Walnut extract (60% ethanol)	*in vivo,* mice	10 and 20 mg/kg, three weeks, PO	- regulated BBB function ↑ mitochondrial function, SOD, ACh, and ATP levels ↓ behavioral dysfunction and memory deficit, MDA level, AChE activity, TNF-α, p-JNK, and IL-1β amounts	(64)
Walnut oil	*in vivo,* mice	10 ml/kg, eight weeks, PO	↑ choline acetyltransferase activity, SOD activity, GSH amount ↓ memory impairment, AChE activity, MDA level in the brain, histological alterations of neurons in CA1 and CA3 regions of the hippocampus	(65)
Walnut	*in vivo,* mice	6% of the daily diet, 24 weeks, PO	↑spatial memory, hydroxy-polyunsaturated fatty acids in the brain ↓arachidonic acid-based oxylipin levels	(51)
Walnut protein hydrolysates	*in vivo,* mice and zebrafish	mice: 333, 666 mg/kg, 21 days, PO zebrafish: 20 mg/l, 7 days	↑ expressions of antioxidant defense-related protein, BDNF, CREB, AChE and Keap1 inhibitors	(100)
Walnut derived peptide (WNP-10)	*in vivo,* mice		-88 differentially expressed proteins in the WNP–10-treated group - regulated phosphatidylinositol-3-phosphate 5-kinase, cathepsin L, N-acetylgalactosamine 6-sulfate sulfatase and AP-3 complex subunit mu-1 expression - maintained lysosome homeostasis ↑ learning and memory capability, phosphorylation of phosphatidylinositol-3-phosphate 5-kinase	(66)
Clinical trials				
Walnut	clinical trial, 708 free-living elders	30–60 g/d, two years, PO	- delayed cognitive decline - no effect on cognition	(67)
Whole walnut	clinical trial, 3632 US adults aged 65 years and older	0·01–>0·08 1 oz. servings per day, 4-year, PO	- neuroprotective effects - positively linked with health behaviors and socioeconomic status ↑ cognitive scores	(68)
Walnut oligopeptide	clinical trial, 36 teenagers and elderly people	170 and 340 mg, 90 days, PO	↑ adult intelligence scale, sleep quality index, average scores for test subjects of English, Mathematics, and Chinese examinations	(69)

ACh: acetylcholine; AChE: acetylcholinesterase; AGEs: advanced glycation end products; ATP: adenosine triphosphate; ATG7: autophagy-related 7; Bax: Bcl-2 Associated X; BBB: blood-brain barrier; BDNF: brain-derived neurotrophic factor; CAT: catalase; DNA: deoxyribonucleic acid; FRAP: ferric reducing antioxidant power; GPx: Glutathione peroxidase; GSH: glutathione; IL-1β: interleukin 1 beta; MDA: malondialdehyde; mRNA: messenger ribonucleic acid; mTOR: mammalian target of rapamycin; NRF2/KEAP1/HO-1: nuclear factor erythroid 2-related factor 2/Kelch-like ECH-associated protein 1/ Heme oxygenase-1; p-CREB: phosphorylated cAMP response element-binding protein; PGE2: Prostaglandin E2; p-JNK: phosphorylated c-Jun N-terminal kinase; PINK1: PTEN-induced putative protein kinase 1; p.o.: oral; ROS: reactive oxygen species; SOD: superoxide dismutase; TNF-α: tumor necrosis factor-alpha

**Table 2:** Effect of *Juglans regia *L. on Parkinson’s disease, depression, anxiety, mood states, pain, and epilepsy

Compound	Study design	Doses/Duration	Results	Ref.
Parkinson’s disease				
Walnut extract	*in vitro,* primary mesencephalic cells *in vivo,* mice	0.1 and 1 µg/ml, 24 hr 100 mg/kg, 6 days, PO	↓ ROS and nitric oxide productions in primary mesencephalic cells - inhibited reduction of striatal dopamine and its metabolites	(73)
Walnut	*in vivo*, mice	6% of the diet, 28 days	↑ dopamine, 3,4-dihydroxyphenylacetic acid, homovanillic acid, ATP in the striatum ↑ GSH, GPx amounts in striatum and substantia nigra, mitochondrial complex I activity in substantia nigra ↓ TBARS, SOD, CAT striatum and substantia nigra, MAO-B activity in the striatum	(71)
Depression, anxiety, and mood states				
Walnut fruit extract	*in vivo*, rats	100 and 150 mg/kg, IP	↓ duration of immobility in the Forced swimming test and tail suspension test	(76)
Different extracts of walnut	*in vivo*, mice	100, 200 and 400 mg/kg, PO	- ethanol extract of *J. regia* showed significant dose-dependent antianxiety and antidepressant activity at 200 and 400 mg/kg	(77)
Walnut	clinical trial, 64 college students	60 g, eight weeks	- improve mood in non-depressed, healthy young males ↑ α-linolenic acid and linoleic acid amount in serum	(78)
Pain				
Aqueous and ethanolic extracts of *J. Regia* L. leaves	*in vivo*, mice	aqueous extract: 0.41, 1.64, and 2.87 g/kg, IP Ethanolic extract: 0.292, 1.17, and 2.044 g/kg, IP	- antinociceptive activity in writhing test - both extracts displayed anti-inflammatory properties	(39)
Methanolic extract of *J. regia* L. leaf	*in vivo*, rats	200 mg/kg, 8 weeks, PO	↑ antioxidant status in the sciatic nerve ↓ degeneration of the sciatic nerves, caspase-3, COX-2, iNOS expression, lipid peroxidation and nociceptive response, blood sugar, behavioral and structural indices of diabetic neuropathy	(83)
*J. regia* L. oil and *J. regia* L. ethyl acetate extract	*in vivo*, mice	12.5, 25, and 50 mg/kg, eight days, PO	↑ serum CAT level ↓ lipid-peroxidation, inflammation, blood glucose, thermal-hyperalgesic and anti-allodynic neuropathic-pain	(84)
Aqueous extract of walnuts	*in vivo*, rodents	5 and 10% of the batches	↓ edema, average abdominal cramps	(85)
Epilepsy				
Walnut kernel	*in vivo*, rats	6% of the diet, two months, PO	↑ seizure threshold ↓ neural death, mortality	(90)
Walnut	*in vivo*, rats	1.2 g, two months, PO	- delayed the kindling procedure ↓ electrical and behavioral parameters of kindling	(91)
Walnut kernel extract	*in vivo*, rats	100 mg/kg, IP	- diazepam and walnut kernel extract presented a synergic anticonvulsant effect ↑ pentylenetetrazole dose required to trigger the first myoclonic jerk ↓ the severity of seizure grades and the mortality rate	(92)
*J. regia* L. fruit ethanolic extract	*in vivo*, mice	200 and 400 mg/kg, PO	↑ the onset of myoclonic jerks dose-dependently, GSH and CAT levels in the brain ↓ MDA level in the brain	(89)
Walnut peptide extracts	*in vivo*, mice	20 mg/kg, IP	- modulated the benzodiazepine receptors ↑ seizure threshold	(88)

ATP: adenosine triphosphate; CAT: catalase; COX-2: cyclooxygenase-2; GPx: glutathione peroxidase; GSH: glutathione; iNOS: inducible nitric oxide synthase; IP: intraperitoneal; MAO-B: monoamine oxidase-B; p.o.: oral; ROS: reactive oxygen species; SOD: superoxide dismutase; TBARS: thiobarbituric acid reactive substances

**Parkinson’s disease**

Parkinson’s disease is a neurological condition that affects about 1% of the elderly population. The loss of dopaminergic neurons in the substantia nigra and a significant decline in striatal dopamine occur in Parkinson’s disease. Moreover, the motor deficits could be caused by reduced dopaminergic activity (70, 71). The development of Parkinson’s disease appears to be influenced by oxidative stress, inflammatory factors, α-synuclein buildup, aquaporin 4, and apoptotic pathways (9, 70, 72) (Figure 4). The oxidative deamination of monoamines, including dopamine, is catalyzed by monoamine oxidase (MAO). In Parkinson’s disease, MAO expression increases. A rise in MAO activity is closely associated with age, and oxidative stress may cause neuronal degeneration in the brain. MAO (specifically MAO-B) is involved in producing ROS, such as hydrogen peroxide, which is harmful to dopaminergic cells and their environment (73).

***In vitro*** **plus** ***in vivo***

The results of an investigation revealed that treating primary mesencephalic cells with walnut extract lowered ROS and nitric oxide production in these cells. Furthermore, administering walnut extract to mice with Parkinson’s disease inhibits the reduction of dopamine and its metabolites in the striatum (73).

***In vivo***

An *in vivo* study indicated that treating mice with Parkinson’s disease resulted in increased dopamine, 3,4-dihydroxyphenylacetic acid, homovanillic acid, and ATP in the striatum; enhanced levels of GSH and GPx in the striatum and substantia nigra; mitochondrial complex I activity in the substantia nigra; decreased thiobarbituric acid reactive substances (TBARS), SOD, and CAT in the striatum and substantia nigra; and lowered MAO-B activity in the striatum (71) (Table 2).

These findings suggest that walnut could be an advantageous therapy option for neurodegenerative diseases like Parkinson’s disease by reducing oxidative stress and preventing the reduction of dopamine and its metabolites in the striatum and substantia nigra. However, more research is needed to confirm it. More research into the method of action, human baseline metabolic rate, BBB permeability, and individual side effects of walnut active substances will be needed in diverse experimental models.

The *in vitro *and* in vivo* studies on the effects of walnut extract on Parkinson’s disease have shown promising results. *In vitro*, the extract was found to reduce ROS and nitric oxide production in primary mesencephalic cells, indicating its potential to mitigate oxidative stress. *In vivo*, administering walnut extract to mice with Parkinson’s disease increased dopamine and its metabolites in the striatum, enhanced anti-oxidant enzyme activities, and decreased oxidative stress markers. These findings suggest that walnut extract could be a therapeutic option for neurodegenerative diseases like Parkinson’s by reducing oxidative stress and preventing the reduction of dopamine and its metabolites. However, further research is needed to confirm these findings and to understand the method of action, human baseline metabolic rate, BBB permeability, and individual side effects of walnut active substances in diverse experimental models.

**Depression, anxiety, and mood states**

Affective disorders, particularly depression and anxiety, are among the most debilitating and common psychiatric complaints. According to World Health Organization epidemiological data, anxiety and depression affect 3.6% and 4.4% of the global population, respectively (74, 75).

***In vivo***

It has been reported that prescribing walnut fruit extract to rats demonstrates its antidepressant properties by attenuating the immobility duration in forced swimming and tail suspension tests (76). It has also been indicated that the administration of an ethanol extract of walnuts to mice resulted in antidepressant and antianxiety activity in mice using the forced swim test and elevated plus maze (77).

**Clinical trial**

A clinical trial assessed the effect of walnut consumption on college students’ mood status, and it was observed that consuming walnuts increased the levels of α-linolenic acid and linoleic acid in serum compared to the placebo group. Additionally, walnuts could considerably improve mood in non-depressed, healthy young males (78) (Table 2).

It might be suggested that adding walnuts to our everyday diet can help alleviate anxiety and depression. More research is required to comprehend the mechanism of the antidepressant activity of walnuts fully. 

**Pain**

The ability of neural systems to perceive threatening or existing tissue injury evolved into the neurophysiological process that generates nociceptive pain. Its supporting task necessitates immediate responsiveness and attention, achieved by inducing the withdrawal reflex, an innate unpleasant sensation, and emotional distress. According to some studies, oxidative stress, inflammation, and apoptosis are the key underlying pain processes (8, 79, 80). Moreover, the International Association for the Study of Pain (IASP) defines neuropathic pain as pain triggered by a somatosensory system lesion or agitation. Hyperalgesia (an amplified reaction to painful stimuli), allodynia (pain in response to stimulants that do not typically cause pain), and dysesthesia (annoying atypical sensation) are all symptoms of chronic neuropathic pain (81, 82).

***In vivo***

Assessing the antinociceptive effects of aqueous and ethanolic extracts of *J. Regia* leaves revealed that the extracts displayed anti-inflammatory properties, and both of them could significantly reduce pain in the writhing test (39). An investigation examined the protective effects of *J. regia* leaf methanolic extract in rats with diabetic nephropathy. The results revealed that the administration of the walnut extract could reduce the nociceptive response and degeneration of the sciatic nerves by attenuating lipid peroxidation as well as caspase-3, cyclooxygenase-2 (COX-2), and inducible nitric oxide synthase (iNOS) expression. It also reduced the blood sugar level (83). Furthermore, it has been reported that administration of *J. regia* oil and ethyl acetate extract to mice with diabetic neuropathy illustrated antinociceptive properties by augmenting serum CAT levels and lowering lipid peroxidation, inflammation, blood glucose, thermal-hyperalgesic, and anti-allodynic neuropathic pain (84). Another* in vivo* study on rodents found that using batches comprising an aqueous extract of walnuts could remarkably reduce edema and average abdominal cramps (85) (Table 2). 

The different extracts of *J. regia* are found to produce antinociceptive effects both centrally and peripherally. These effects could be mediated by non-opioid receptors or suppression of the cyclooxygenase enzyme, as well as the anti-oxidant and anti-apoptotic properties of the extracts. The extracts were also found to have anti-inflammatory properties in both acute and chronic phases.

**Epilepsy**

One of the most common neurological conditions affecting millions of individuals worldwide is epilepsy (86). The medication used for treatment is frequently pharmacological, primarily affecting ion channels and neurotransmitter receptors (87). Despite the antiepileptic medications that are already available, about 30% of epileptic patients still experience seizures that seem to be resistant to all pharmacological approaches (88). The brain’s oxidant and anti-oxidant systems become unbalanced during seizures, leading to lipids, proteins, and DNA oxidation, which results in neurodegeneration (89).

***In vivo***

As the results of an investigation showed, adding walnut kernels to rats’ diets increased seizure threshold and decreased neural death and mortality after the injection of pentylenetetrazole (90). The findings of another study indicated that walnut consumption could postpone the kindling procedure and reduce the electrical and behavioral parameters of kindling (91). Moreover, it has been reported that prescribing walnut kernel extract to rats before pentylenetetrazole injection significantly increased the required pentylenetetrazole dose to initiate the first myoclonic jerk while decreasing the severity of seizure grades and the mortality rate. Furthermore, diazepam and walnut kernel extract presented a synergic anticonvulsant effect (92). It has been observed that prescription of *J. regia* fruit ethanolic extract before injection of pentylenetetrazole in mice resulted in enhanced initiation of myoclonic jerks dose-dependently, increased GSH and CAT levels, as well as attenuated MDA levels in the brain (89). Besides, intraperitoneal injection of walnut peptide extracts was seen to modulate the benzodiazepine receptors and increase seizure threshold (88) (Table 2).

The findings from *in vivo* studies demonstrate the potential of walnut consumption and its derivatives in mitigating seizure activity and enhancing seizure threshold in animal models of epilepsy. Walnut kernels, when incorporated into rats’ diets, have been shown to increase seizure threshold and decrease neural death and mortality after inducing seizures. Additionally, walnut consumption has been found to postpone the kindling procedure, reduce the electrical and behavioral parameters of kindling, and increase the required dose of pentylenetetrazole to initiate the first myoclonic jerk, indicating an anticonvulsant effect. Moreover, walnut kernel extract administered before inducing seizures resulted in decreased severity of seizure grades and mortality rates, with a synergistic anticonvulsant effect observed when combined with diazepam. Ethanol extracts from *J. regia* fruit were also found to enhance seizure initiation dose-dependently while increasing antioxidant levels and reducing oxidative stress in the brain. Furthermore, walnut peptide extracts were shown to modulate benzodiazepine receptors and increase seizure threshold. These findings suggest that walnut-derived compounds possess anticonvulsant properties and may have therapeutic potential in managing epilepsy, particularly for drug-resistant seizures. Further research is needed to elucidate the underlying mechanisms and explore their clinical applications in humans with epilepsy.

## Conclusion

The neuroprotective effects of *J. regia*, commonly known as walnut, have been extensively investigated through various studies. The results of these studies suggest that the consumption of *J. regia* or its active compounds, including polyphenols, flavonoids, and omega-3 fatty acids, can help in the prevention and treatment of neurological disorders such as Alzheimer’s disease, Parkinson’s disease, depression, anxiety, epilepsy, and pain by various underlying mechanisms, for instance, anti-oxidant, anti-inflammatory, and anti-apoptotic effects. While further research is needed to fully understand the mechanisms of action of *J. regia* and its potential therapeutic applications, the available evidence highlights the potential of this nut as a natural and safe neuroprotective agent. 

It is also important to note that studies on herbal medicines should be taken into account because many aspects of their safety and effectiveness are still unclear. Additionally, more studies are required to evaluate the safety and efficacy of various walnut constituents in treating various nervous system diseases. Moreover, a combination of physiological, pharmacological, and pharmacokinetic approaches must be used to examine multi-component walnut mixtures’ potential antagonistic and synergistic effects. The discoveries may contribute to the future expansion of the therapeutic benefits of *J. regia* as well as its use in modern medicine. 
